# Does the Diabetes Specialist Nursing workforce impact the experiences and outcomes of people with diabetes? A hermeneutic review of the evidence

**DOI:** 10.1186/s12960-019-0401-5

**Published:** 2019-08-07

**Authors:** Jessica Lawler, Paul Trevatt, Clare Elliot, Alison Leary

**Affiliations:** 1 London South Bank University, School of Health and Social Care, 103 Borough Rd, London, SE1 0AA United Kingdom; 2 Cardiovascular Disease/End of Life Care, Clinical Networks, NHS England (London region), London, United Kingdom; 3 South West London Health & Care Partnership, London, United Kingdom

**Keywords:** Clinical Nurse Specialist, Diabetes, Workforce, Inpatient diabetes care, Safety, Medication error, Education, Hermeneutics

## Abstract

**Objectives:**

The aim of the hermeneutic review was to identify and clarify the mechanisms by which the Diabetes Specialist Nursing workforce affect the outcomes of diabetes patients, with a focus on those in the United Kingdom. A clarification of diabetes specialist nurses’ work is necessary in understanding and improving diabetes inpatient care.

**Design:**

The design is a hermeneutic evidence review and was part of a wider evaluation of Diabetes Inpatient Specialist Nurses for which the evidence was sourced. The literature search was limited to specialist nursing workforce caring for adults with diabetes. In order to gain global understanding of the impact of specialist nursing in diabetes, worldwide literature was included.

**Methods:**

A hermeneutic literature review of 45 publications was carried out, which included citation analysis. Relevant literature was identified from 1990 to 2018.

**Results:**

Evidence suggests that Diabetes Specialist Nurses educate patients and other healthcare professionals as well as delivering direct care. The outcomes of these actions include a reduced patient length of stay in hospital, reduced inpatient harms and complications, and improved patient satisfaction. Additionally, they are cost-effective.

**Conclusions:**

The Diabetes Specialist Nursing workforce is essential in diabetes care, particularly in hospital settings. They improve patient experience and outcomes.

**Electronic supplementary material:**

The online version of this article (10.1186/s12960-019-0401-5) contains supplementary material, which is available to authorized users.

## Introduction

Diabetes in the United Kingdom is common: almost 3.7 million people are currently diagnosed with diabetes, representing 1 in 15 of the United Kingdom population. Around 90% of those have type 2 diabetes and 10% have type 1. It is on the rise; almost 100,000 people were diagnosed with diabetes from 2016 to 2017 [1]. Diabetes costs the NHS (National Health Service) around £10 billion a year in hospital admissions of people with diabetes (PWD) and its complications [2].

An estimated 1 in 6 United Kingdom hospital inpatients have diabetes [3]. It is not uncommon for PWD admitted to hospital (for diabetes or non-diabetes related reasons) to have reduced overall control of their condition, with insulin treatment, timing of meals and glucose monitoring affected [4]. It is widely understood that inpatients who suffer adverse events and medication errors experience an increased length of stay of between 2 to 8 days [5]. The need for quality improvement in inpatient diabetes care has been highlighted [6].

Diabetes UK (a large UK diabetes charity), Trend UK (working group of diabetes nurses) and the Royal College of Nursing’s (RCN) position statement on Diabetes Specialist Nurses (DSNs) state that ‘DSNs are cost effective, improve clinical outcomes and reduce length of stay in hospital.’ [2]. They also recognise that they are greatly undervalued, and condemn the stagnation of DSN numbers [2]. Currently, the number of Diabetes Inpatient Specialist Nurses (DISNs) is significantly lower than recommended levels in the United Kingdom. The National Diabetes Inpatient Audit (NaDIA) [3] reported that 28% of hospitals had no DISNs. There is no national census of DSN posts, unlike other specialism areas, such as cancer [7]. Therefore, it is challenging to identify where inequalities exist and if there are variations by geography or trust size. It is also impossible to predict the future need of the specialist workforce.

The 2016 target was for ‘all hospitals and trusts to provide a Diabetes Inpatient Specialist Nurse service, with at least one DISN per 250 patient beds’ [8]. NICE Quality Standards for diabetes care for adults recommend that all hospitals should implement a DISN service [9] and NaDIA recommends that this should be a 7-day provision [3]. However, as of 2017, only 8.8% of sites in England and Wales provide a 7-day DISN service.

Diabetes Specialist Nurses provide complex care for PWD across a broad range of settings. This includes clinical care, education and psychological care of patients and healthcare professionals, which requires high levels of expertise [10]. DSNs in the United Kingdom are defined by Diabetes UK as ‘registered nurses who are clinical experts in diabetes and work exclusively in diabetes care’; however, at present, there is no title protection or national accreditation framework for DSNs. This has resulted in a wide variation in DSNs’ credentials and roles [10]. The Diabetes Specialist Nursing workforce survey [10] illustrates the need for more DSNs to support the increase in diabetes prevalence. Clinical nurse specialists (CNS) in the United Kingdom work at advanced levels with increasing autonomy. There is a large body of evidence that links the positive impacts of clinical nurse specialists with patient outcomes, symptom control and effective liaison and training with other professionals [11]. CNS are a workforce that is widely undervalued and under scrutiny, having to repeatedly prove their worth. Often their roles are described and defined in oversimplified terms that fail to communicate the breadth and complexity of the work they undertake [12].

There is no ideal mix of health care professionals’ skills universally; however, reviewing existing practices is necessary for informed decision-making [13]. A review of literature is necessary to clarify the ways in which this workforce address the issues that exist in diabetes inpatient care, currently and over time. This hermeneutic review of evidence discusses the ways in which DSNs impact diabetes inpatients’ outcomes, and would support an increased provision of their services.

### Aims

The aim of this review was to identify and clarify the mechanisms by which the Diabetes Specialist Nursing workforce affect the outcomes of people with diabetes. The questions of this literature review are detailed in Table 1.

**Table 1:** Literature review questions

Literature review question	
1. What are the interventions DSNs undertake with diabetes inpatients?	
2. What are the outcomes of DSN’s interventions with diabetes inpatients?	
3. Do DSN’s actions improve inpatients’ outcomes and hospital experiences?	

## Methods

**Fig. 1:**
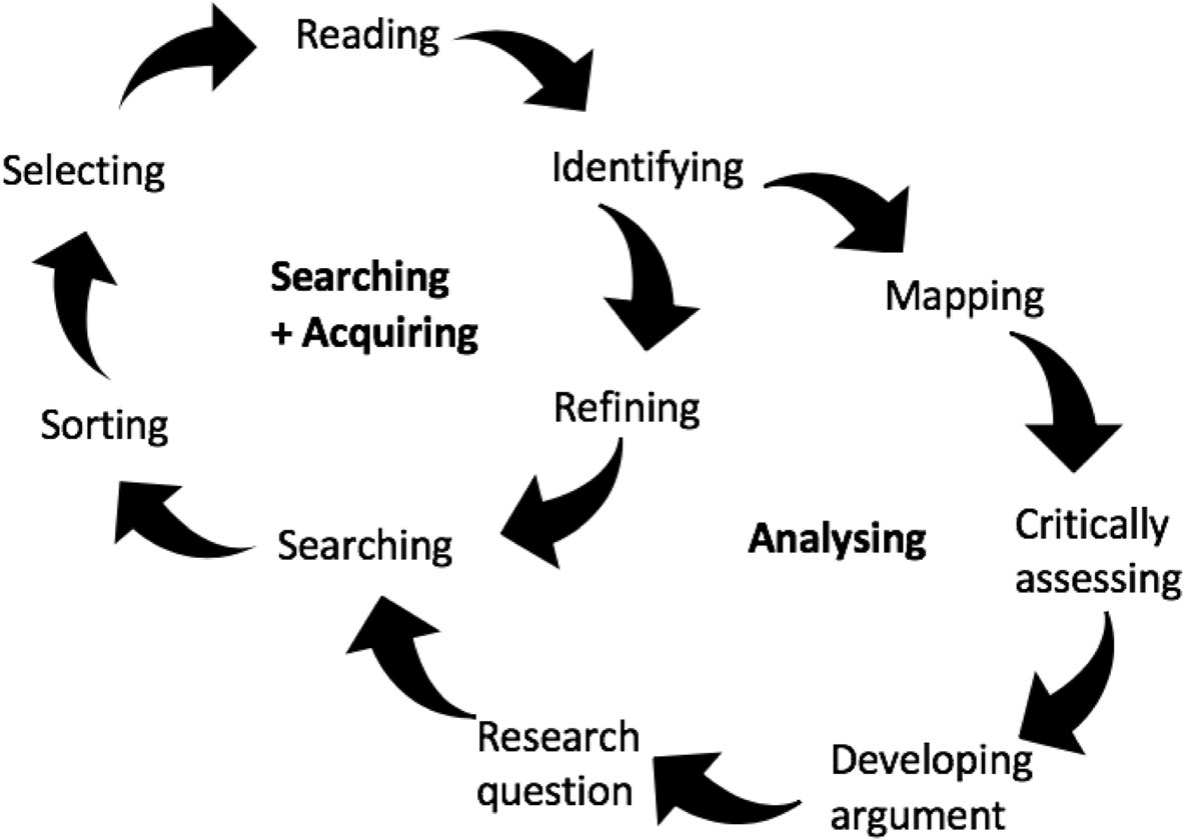
The hermeneutic circle as a framework for reviewing literature, adapted from Boell and Cecez-Kecmanovic [14]

A hermeneutic approach [14] was taken to review the literature in the area (Fig. 1). The goal of a hermeneutic review is to develop a better understanding of a topic area in an iterative manner [14, 15]. Hermeneutic review is suited to uncovering the work of DISNs due to its focus on context and surfacing meaning from lived experience. It is an interpretative method of reviewing multiple different sources of evidence, questioning the literature and remaining open to what could be revealed [16]. The hermeneutic approach is based on the question of human understanding as developed by Heidegger and Gadamer [14, 17]. It allowed for a wide perspective and analysis of a broad body of evidence.

An initial exploratory search of literature was carried out in May 2018 on a number of databases: details and search terms are in Table 2. All search terms were in English. The initial search generated 8697 items.

**Table 2:** Literature search terms and databases

Search terms	
Diabet* AND specialist nurse OR advanced practice OR clinical nurse specialis* OR expert practitioner OR advanced nurse practitioner AND Patient education OR patient self care OR patient self-care OR patient rescue AND Staff educat* OR staff train*	
Databases searched	
AMED CINAHL PubMed MEDLINE	

### Inclusion and exclusion criteria

This review was part of a wider evaluation of DSNs in the United Kingdom: it was limited to diabetes specialist nursing in adults, as this was the scope of the project and provided the background to this area of enquiry. However, literature focussing solely on inpatient diabetes nursing is limited, and DISNs are often written about within broader papers on DSNs. Therefore, broader papers on DSNs were included and abstracts were analysed to establish relevancy to the review questions. Search parameters were limited from 1990 to 2018 in order to encompass seminal papers which focus on the direction of the DSN role and to understand the evolution of the role, in particular as an educator in hospital settings. Results were limited to peer-reviewed papers only. This delimited the results to 545 papers.

After abstracts were established as relevant, papers were read, re-read and analysed as in the hermeneutic approach [14]. Citations were analysed, and snowballing (also known as reference tracking: using references to identify additional papers of relevance) was used to expand the search of literature. Understanding of individual texts was built in the context of the whole body of literature as described by the hermeneutic circle [14]. In total, including those identified through snowballing, 45 publications were identified as relevant and specific to the review questions, including grey literature, academic papers, reports, position statements and NaDIA. Further details on these papers and their relevance to review questions can be found in Additional file 1. The hermeneutic circle was broken and left at the search stage, when a point of saturation had been reached [14]. This is when additional papers make only a marginal contribution to further understanding [14]. The themes that emerge here are not exhaustive but provide a break in the cycle of searching and analysis. It is recognised that many of these themes can be further explored and expanded upon.

## Results

A number of themes emerged from the review of evidence. These were identified by the authors from recurrent topics in the literature, framed around the findings from successive NaDIAs. The themes have been separated into the actions DSNs take: patient education, staff education, direct patient care, psychological care and the outcomes of DSN interventions—a reduction in inpatient harm and length of stay and improved patient satisfaction. These themes are divided in this way as a reflection of the literature review questions which centred around the interventions and the outcomes of DSNs’ interventions. Challenges in practice will be identified and discussed.

### Actions of DSNs

#### Patient education

Diabetes Specialist Nurses play an increasingly crucial role in educating patients and healthcare professionals [18]. Evidence suggests that in 97% of services in the United Kingdom, DSNs provide education to people with diabetes and staff in both primary and secondary care settings [19].

Much of the education given by DSNs to patients is with a view to enable self-management of diabetes at home. This education includes advice on medicine administration, for example, using insulin pumps and measuring blood glucose levels. Education most frequently occurs in one-to-one educative conversations with the patient, or additionally with family members or caregivers [20].

Self-management of diabetes is a challenging and complex process for patients: physically, emotionally, socially and intellectually. Patient education and self-management is essential in diabetes, combined with support from family and peers. It is widely accepted that self-management of diabetes can promote quality of life [21]. Kousoulis’ realist review of over 5,500 individuals in six European countries showed a distinct policy shift towards patient-centred self-management of diabetes in primary care [21].

‘Patient education is the cornerstone of diabetes management’ according to Feddersen and Lockwood [22]. DISNs play a pivotal role in educating patients in hospital and empowering patient self-management of their diabetes [23]. Care and advice given by DSNs in addition to standard care has resulted in increased patient knowledge and confidence [24]. This is essential for positive patient outcomes: patient confidence can delay complications, reduce hospitalisations, facilitate discharge and prevent readmission [20]. Evidence suggests that education in controlling glycaemia has sustained benefits and translates to reduced associated micro and macro cardiovascular risk factors [25]. Conversely, Kousoulis’ [21] realist review reported that diabetes education should be regarded to have a broadly positive effect on patient outcomes, but a long-term benefit on glycaemic control cannot be assumed or expected.

Timing and location is important: patient education is particularly critical for newly diagnosed PWD [26] and in hospital settings [27]. According to Davies and Davis [28], education is essential for diabetes inpatients to ‘make informed choices about their self-care’. Furthermore, disempowerment in United Kingdom hospitals has been regularly noted [29]. In James et al.’s study [18] of 159 United Kingdom diabetes centres, 97% of services provided patient education sessions.

#### Professional education

The delivery of appropriate care relies on adequate staff knowledge and skills, and successful coordination and cooperation between health care professionals. According to Ross et al. [20], diabetes specialists bridge gaps in expertise and knowledge across teams by providing staff with specific diabetes education. They increase both patient and staff self-efficacy which is connected with reduced length of stays (LOS). DSNs proactively identify and anticipate cases that need specialist attention. They respond reactively and prevent problems escalating early.

Carey et al. [5] suggest that health care professionals’ education is a contributing factor to medication and prescription errors in diabetes and that DSN prescribers can reduce these errors through regular patient assessment and review and individual education sessions with medical and nursing staff. Additionally, NHS England [30] (2016) suggests that DISNs reduce time requirements on other clinical staff due to effective and efficient management.

Due to disease complexity and new advancements in treatment, it is apparent that generalist staff cannot be expected to maintain diabetes expertise. This highlights the necessity of DSNs, who can advise and guide other staff members [22]. A report on all identifiable diabetes centres in the United Kingdom in 2007 [18] found that in 159 centres, 76% of DSNs deliver education sessions to both healthcare professionals and patients. Hospital DSNs provided the most education for healthcare professionals compared to community DSNs, Nurse Consultants and Paediatric DSNs, with 94% of hospital DSNs delivering professional education. How this education is delivered to health care professionals is not explicitly defined and has been purported variedly, ranging from corridor conversations to structured education sessions [18].

Kousoulis et al. [21] found that PWD often report receiving inconsistent or contradictory advice from different health care professionals. Additionally, Carey et al. [5] suggest that staff lack of knowledge and misperception of patients’ cases contributes to increased medication errors in hospital. These, as will be later detailed, can be reduced by DSNs.

Conversely, the presence of specialist diabetes teams has reportedly had some unintended consequences. Ross et al. [20] found an erosion of ward staff skills with the introduction of a specialist diabetes team. This created delays in patient treatment and often over-burdened the specialist nurses. This could perhaps be countered by further education and empowerment of ward staff to ensure they maintain the necessary diabetes confidence and knowledge to respond.

#### Direct patient care and medicines management

Hospital DSNs provide direct inpatient care [28]. One of DSNs’ primary responsibilities is managing patient treatment recommendations. DISNs have a crucial role in improving glycaemic control in patients in hospital. Evans et al. [31] describe this role as ‘vital’. Patient care provided by inpatient diabetes specialist teams is efficient. The national ‘Think Glucose’ initiative launched in 2008 utilises a ‘traffic light’ system to guide which patients should be referred to the specialist team [32]. This system, covering over 30 ‘red’, ‘amber’ or ‘green’ cases ensures that patients that need to be seen are being seen by a diabetes specialist, whilst also ensuring effective use of resources and DISN time.

Direct diabetes problems and complications because of diabetes include foot ulceration, renal failure, diabetic retinopathy, ischaemic heart disease, stroke and peripheral vascular disease. These complications are often the reasons for diabetes patient admission to hospital [33]. Eighty-nine percent of DSNs deliver nurse-led clinics for patients with these complications [10].

Additionally, DSN telephone consultations enable outpatients that require follow-up after discharge to receive care. Evidence suggests that as of 2009, in 71% of hospital-based services, telephone helplines were offered to PWD [19]. These were operated by hospital DSNs in 94% of cases. This ‘telemedicine’ reportedly improves ongoing diabetes care, reduces the number of acute hospital admissions and is cost-effective [31].

With advancements in technology playing an ever more important role in healthcare, DSNs also facilitate digital health care into diabetes treatment. Their role in enhancing the accessibility and convenience of diabetes care is well recognised [34].

DSNs intervene in medicines management, which reportedly has a positive effect on the delivery of medicines [5]. Their expertise is crucial in adjusting treatment and managing comorbidities, in particular during hospital stay. The reduction of glycosylated haemoglobin (HbA1c) as a primary outcome measure has been used in a variety of studies on DSN’s effectiveness in patient health. Loveman et al. [35] found that specialist nurses were effective in reducing this in the short-term, with the long-term not being fully studied.

Ross et al. [20] reported that DSNs make more knowledge-based decisions and have more holistic views of patients than their counterparts. They also suggest that DSNs make higher-level decisions, and rather than simply following protocol, they take the clinical complexity of each case into consideration. DSNs are also reported to be responsible for the coordination of multidisciplinary responses to complex cases, in particular, in organising care plans. In this way, DISNs provide ‘continuity of care’ [26].

A report of specialist diabetes services in 2007 found that 49% of hospital DSNs are involved in prescribing [18]. That is compared to the estimated 5% of the total United Kingdom nursing workforce in 2016 qualified as nurse independent or supplementary prescribers [36]. However, the necessity of DSNs to prescribe has been contested. A comparison of prescribing and non-prescribing nurses in diabetes patient management in general practice found little difference in patient outcomes in self-care and HbA1c levels [37]. However, there were significantly higher levels of satisfaction among prescribing nurses’ patients, and blood glucose testing was more prevalent in this group. This may have been down to longer consultations, enabling extra advice and information exchange. As not all DSNs prescribe, this can often cause delay in treatment [20].

#### Psychological care and counselling

DSNs are often seen as a pillar of knowledge during hospital stays and in primary and secondary care alike. Loveman et al. [35] conducted an intervention review where they found that patients often contact their DSN in preference to their GP. This review was of six trials including 1382 participants followed for six to twelve months. DSNs provide emotional, psychological and social care for both patients and their families [38]. Loveman et al. [35] reported that ‘patients in contact with specialist nurses are generally satisfied with the level of care that they receive’.

This specialist role is known to have a positive effect on diabetes patients’ outcomes [5]. DSN’s role is crucial for patients and families to establish trust and confidence in health care providers and for health promotion to be maximised [38]. Additionally, DISNs are suggested to bridge communication gaps between clinical partners and patients, acting as an intermediate role that can prevent problem escalation [20].

A diabetes patient experience study at Derby Hospital Foundation Trust revealed feelings of fear and anxiety at the prospect of visiting hospital, and ‘feelings of loss of control, concerns that the staff did not have sufficient expertise in diabetes, and a lack of knowledge in the care to expect whilst in hospital.’ [32]. It is evident that psychological care and advice is necessary for PWD, especially in inpatient settings.

### Outcomes of interventions

#### Reduction of inpatient harm

Due to the complexity of diabetes, medication errors are common in inpatients. NaDIA 2017 [3] reported that 31% of patients in the audit had experienced at least one diabetes medication error whilst in hospital. These included both prescription errors and medication management errors, and are much higher than errors in other diseases in hospital. As of 2014, there was an average prescribing error rate in United Kingdom hospitals of 7% and a rate of 3–8% of medicine administration [39]. NHS England [30] asserts that DISNs reduce inpatient harms by reducing medication errors and hypoglycaemic events. NaDIA 2016 [8] highlighted the need for healthcare professionals to have the knowledge, experience and confidence in managing diabetes medication to reduce medication errors. Additionally, Diabetes UK, Trend UK and RCN’s 2014 [40] position statement presented evidence that suggests that DSNs, especially those with Nurse Prescribing skills, significantly reduce insulin error, with a consequential reduced LOS.

NaDIA 2017 [3] revealed that around 1 in 25 inpatients with type 1 diabetes develop diabetic ketoacidosis (DKA) and around 1 in 800 inpatients with type 2 diabetes develop hyperosmolar hyperglycaemic state (HHS) during their hospital stay. These hospital-acquired emergency states are extremely serious, potentially fatal and are preventable conditions that should not occur during hospital admission. Diabetes specialist teams with sufficient knowledge, capacity and expertise are crucial in reducing inpatient harms such as DKA and HHS by using specific and tailored medicines management [20].

Carey et al. [5] found a significant reduction in the number of medication errors with a DSN study group and an overall positive effect on medicine delivery systems. Their study looked at inpatient care of a convenience sample of 56 diabetes patients over 8 months. Thompson et al. [41] suggest that insulin adjustment according to advice from a diabetes nurse educator is effective in improving glucose control in diabetic patients. Additionally, Vissarion et al. [38] suggest that DSNs play a crucial role in responding to crises.

Increasing workload and staff cuts are affecting patient care. With demand for diabetes services rising without increasing DSN numbers, 78% of DSNs have concerns that their workload is impacting on patient care and/or safety [10].

#### Reduction of length of hospital stay and prevention of hospital admissions

It has been well documented that a DISN service can help reduce the length of hospital stay for people with diabetes [23, 26, 42]. NHS England 2016 [30] asserts that a DISN (1 nurse per 250 inpatient beds) will reduce LOS for diabetes inpatients. This is widely supported by the literature: Cavan et al. [43] found that the introduction of a ward-based diabetes nurse advisor was associated with significant reductions in LOS. Carey et al. [5] found that the median LOS of diabetes patients was reduced by three days by the presence of the DSN prescriber; analysis was made pre and post intervention by the nurse. Additionally, the savings in costs were enough to finance the post. Alabraba et al. [26] reported that a DISN team ensured timely and appropriate discharge and follow-up.

Mahaffey et al. [44] suggest that early reviews of diabetes patients by DISNs in Accident and Emergency (A&E) would prevent hospital admissions. Their study of over 3.5 years found a significant number of people attending A&E were able to be treated and discharged home without hospital admission. They estimated savings of £35,000 over 3.5 years at their service through reduced bed occupancy and patient-focused care.

Davidson et al. [45] found a demonstrably lower hospital resource use for patients under DSN care and significantly fewer emergency room visits and hospitalisations for preventable diabetes-related causes. Sampson et al. [42] also found that diabetes excess bed occupancy was notably reduced by the introduction of a DISN service in a study of 6 years.

As previously discussed, DISNs play a critical role in patient education and promotion of patient self-management. Feddersen and Lockwood’s [22] findings suggested that a greater awareness and knowledge of diabetes in both patients and staff can result in a shorter hospitalisation. Educational programs provided by Inpatient Diabetes Educators have been directly linked to a decrease in readmissions.

#### Increased patient satisfaction

DSNs have been associated with increased patient satisfaction. Courtenay et al. [37] reported an increase in diabetes patient satisfaction when consulted by a prescribing nurse, due to increased consultation time, and establishing relations between nurse and patient. Their study was of 214 patients in the United Kingdom. In Taylor’s [46] study of 169 patients, 92% reported that a DSN led care management programme was moderately to extremely helpful in preparing them to manage their condition. Cavan et al. [43] also found that a DSN-led programme of care for newly diagnosed type 2 diabetes patients was clinically effective with high levels of patient satisfaction and motivation. In workshops aimed at driving improvements in diabetes care and patient experience in London, people with type 1 diabetes reported that they value and would like to see more education and support for family and friends. People with type 2 reported that they would like to see more person-centered care and be seen by the same person [47]. The evidence suggests that DSNs improve patient satisfaction through more personal and specific consultations of longer lengths, educative sessions and empowerment to self-manage.

#### Challenges in practice

Due to resource limitations and staff scheduling, specialist nurses are not always available to inpatients, particularly at weekends and out-of-hours. This results in ward staff taking full responsibility for diabetes inpatient’s complex care [20]. With the increasing number of diabetes inpatients, there are restraints in DISNs’ capacity. These restraints have in some cases been alleviated by the introduction of a Diabetes Clinical Assistant post to support the Diabetes Specialist team [48].

Evidence has demonstrated that DSNs have an increasingly limited access to professional development and opportunity for study leave and research. This suggests that DSNs’ skill and knowledge development faces challenges [1, 10]. This has been described as ‘concerning’ and combined with a lack of long-term job security, could result in recruitment and retainment difficulties [18].

## Discussion

From this review, the role of the DSN workforce in improving the experience of patients and clinical outcomes is critical and varied. This review has elucidated some of the main mechanisms by which DSNs achieve this. Evidence suggests that DSNs reduce patient length of stay in hospital [49], reduce inpatient harms and complications [30], educate patients and other health care professionals [18], and improve patient satisfaction [37]. In this review, DSN’s actions, and the results of DSN’s actions have been reported in separation. This was for ease of reading and understanding of the role versus the role’s outcome. However, these two are of course connected and the actions do have influence on the outcomes.

There is a profound mismatch between DSN workforce’s capacity and the steadily rising demand for diabetes services [10, 29]. One in 15 of the total United Kingdom population have diabetes (7%) [3]. Under current trends, the number of people in the United Kingdom with diabetes is expected to increase to 5.2 million by 2025 [10]. As there is no current United Kingdom workforce data recording the total number of DSNs it is impossible to accurately predict how many specialist nurses will be required to meet the needs of the rising tide of patients. There may be value in carrying out a census of all DSNs to support workforce planning.

Access to high quality integrated care at the right time is essential for successful diabetes management. DSNs are an integral and indispensable part of ensuring effective diabetes care in hospital settings. DSNs have been identified as the first point of contact for some diabetes inpatients [35]. This is a profound finding and could perhaps illustrate some patients’ views of DSNs as providing therapeutic relationships and having expert advocacy. They have a large role in decision making and care planning that can instil confidence in patients and motivation to self-manage [21].

Evidence shows that DISNs are cost-effective [50]. NHS England 2016 [30] goes as far to say that expanding DISN services will result in financial savings, from reduced LOS and fewer inpatient harms. This is increasingly relevant as the NHS in England alone spends an estimated £2.3–£2.5 billion a year on inpatient care for PWD: approximately 11% of total NHS expenditure on inpatient care [50].

One area of contention is that of educating patients in hospital settings. Some research suggests that bedside education from specialist nurses is inappropriate, as patients are too ill to learn [51]. Whether this is an inefficient method of education or perhaps an essential part of healthcare delivery is an area that this review has not covered, and that merits further exploration.

The importance of the DSN workforce in diabetes care is evident. The implications of this literature review on practice are to iterate the necessity to protect and propagate the diabetes specialist nurse workforce in the United Kingdom.

### Strengths and limitations

This review clarifies and details the role and impact of Diabetes Specialist Nurses, a role that lacks a national accreditation framework and title protection in the United Kingdom. It offers breadth and synthesis of knowledge, it is of worldwide literature. The limitation in literature fields only allows some depth to the analysis. There is a risk of oversimplifying the literature at this scale.

## Conclusion

A clarification of DSNs’ role is necessary for understanding and improving diabetes inpatient care. The literature reviewed here would support a continued and increased provision of diabetes specialist nurse services in the United Kingdom. DISNs have a diverse and expanding role that is critical to diabetes care. This review has revealed the mechanisms by which DSNs impact diabetes patients’ experiences, with a focus on those in hospital. These methods include education of patients and professionals [18], direct patient care and reducing inpatient harms [30], reducing length of stay and hospital admissions [49], and an increase in patient satisfaction [37].

## Additional file

Additional file 1: Papers reviewed and review questions. (DOCX 31 kb)

## Data Availability

All data generated or analysed during this study are included in this published article. All of the papers reviewed are in the public domain.
