# Supplementary material for: Does the Diabetes Specialist Nursing workforce impact the experiences and outcomes of people with diabetes? A hermeneutic review of the evidence
Source: Hum Resour Health. 2019 Aug 7;17:65. doi: 10.1186/s12960-019-0401-5 (PMC6686398; doi:10.1186/s12960-019-0401-5)
Supplement: Supplementary file 1 — Papers reviewed and review questions. (DOCX 31 kb) [file 12960_2019_401_MOESM1_ESM.docx]

**Additional File 1.**

Additional Table S1. Papers reviewed and review questions

|  |  | Review Questions | | |
| --- | --- | --- | --- | --- |
|  | **Country** | **What are the interventions DSNs undertake with diabetes inpatients?** | **What are the outcomes of DSN’s interventions with diabetes inpatients?** | **Do DSN’s actions improve inpatients’ outcomes and hospital experiences?** |
| 1. Alabraba V, Floyd E, Wallymahmed M. “Delivering a diabetes inpatient specialist nursing service: The Aintree experience” *Journal of Diabetes Nursing* Vol 14 No 10 2010 | UK | X | X | X |
| 1. Bonney R and Crowe S, “Outcomes that matter” A summary report of outcomes that matter to people with type 1 and type 2 diabetes. London Diabetes Clinical Network, Professor Sir Muir Gray's Oxford Centre for Triple Value and Diabetes UK December 2018 | UK |  | X |  |
| 1. Brown S. “Effects of educational interventions in diabetes care: a meta-analysis of findings.” *Nursing Research* 1998;37 (4):223–30. | Global | X |  |  |
| 1. Carey N, Courtenay M, James J, Hills M, Roland J. “An evaluation of a Diabetes Specialist Nurse prescriber on the system of delivering medicines to patients with diabetes.” *J Clin Nurs*. Jun;17(12):1635-44. 2008 | UK | X | X |  |
| 1. Cavan D, Hamilton P, Everett J, Kerr D. “Reducing hospital inpatient length of stay for patients with diabetes.” *Diabetic Medicine* 2001;18(2):162–4 | UK |  | X |  |
| 1. Courtenay M, Carey N, Gage H, Stenner K, Williams P. “A comparison of prescribing and non-prescribing nurses in the management of people with diabetes.” *J Adv Nurs.* 2015 Dec;71(12):2950-64. | UK |  |  | X |
| 1. Courtenay M. “An overview of developments in nurse prescribing in the UK.” *Nursing Standard.* 2018 | UK | X |  |  |
| 1. Daultrey H, Gooday C, Dhatariya K. “Increased length of inpatient stay and poor clinical coding: audit of patients with diabetes” *RSM Short* Rep. Nov 2011; 2(11): 83. | UK | X |  |  |
| 1. Davidson M, Ansari A, Karlan V. “Effect of a Nurse-Directed Diabetes Disease Management Program on Urgent Care/Emergency Room Visits and Hospitalizations in a Minority Population” *Diabetes Care* 30(2), Feb 2007 | US |  | X | X |
| 1. Davies M and Davis R. “Role of the hospital diabetes specialist nurse: perception vs reality” *Journal of Diabetes Nursing* Vol 2 No 4 1998 | UK | X |  |  |
| 1. Davies M, Dixon S, Currie CJ, Davis RE, Peters JR. “Evaluation of a hospital diabetes specialist nursing service: a randomized controlled trial.” *Diabet Med.* Apr 2001;18(4):301-7. | UK | X | X | X |
| 1. Diabetes UK “The Cost of Diabetes Report” Diabetes UK, London. Jan 2014 | UK |  | X | X |
| 1. Diabetes UK “Collation of Inpatient Experiences.” Diabetes UK, London. 2007 | UK | X |  |  |
| 1. Diabetes UK “Diabetes Prevalence 2017” Diabetes UK, London. November 2017 | UK | X |  |  |
| 1. Diabetes UK “DIABETES SPECIALIST NURSING 2016 WORKFORCE SURVEY: A workforce in crisis” Diabetes UK, London. | UK | X |  |  |
| 1. Diabetes UK, Trend UK, Royal College of Nursing. “Diabetes Specialist Nurses: Improving Patient Outcomes and Reducing Costs, Position Statement.” Feb 2014 | UK |  | X | X |
| 1. Eaglesfield, B “ThinkGlucose at an acute hospital – a “roller-coaster” project.” *Journal of Diabetes Nursing* Vol 16 No 9 2012 | UK | X |  |  |
| 1. Evans, N. R., Richardson, L. S., Dhatariya, K. K. and Sampson, M. J. “Diabetes specialist nurse telemedicine: admissions avoidance, costs and casemix.” *Eur. Diab. Nursing,* 9: 17-21. 2012 | UK | X | X | X |
| 1. Feddersen E, Lockwood DH. “An inpatient diabetes educator’s impact on length of hospital stay.” *Diabetes Educator* 1994;20(2):125–8. | US | X | X |  |
| 1. Flanagan D, Moore E, Baker S, Wright D, Lynch P. “Diabetes care in hospital—the impact of a dedicated inpatient care team” *Diabetic Medicine,* Volume25, Issue2, Pages 147-151, February 2008 | UK |  | X |  |
| 1. Gardiner, F, Nwosea E, Bwititic P, Crocketta J, Wang L. “Does a hospital diabetes inpatient service reduce blood glucose and HbA1c levels? A prospective cohort study” *Annals of Medicine and Surgery* 26, 2018 15–18 | Australia |  | X | X |
| 1. Gosden C, James J, Winocour P, Turner B, Walton C, Nagi D, Williams R, Holt R. “Leading the way: The changing role of the diabetes specialist nurse” *Journal of Diabetes Nursing* Vol 13 No 9 2009 | UK | X |  |  |
| 1. Hardy D and Stanisstreet D. “Is it time for a review of the skill mix within the diabetes inpatient specialist nurse team?” *Journal of Diabetes Nursing*, Vol. 21 Issue 5, p172-173, 2p. 2017 Publisher: SB Communications Group, A Schofield Media Company. | UK | X |  |  |
| 1. James J , Gosden C, Winocour P, Walton C, Nagi D, Turner B, Williams R and Holt R. “Diabetes specialist nurses and role evolvement: a survey by Diabetes UK and ABCD of specialist diabetes services 2007” Journal compilation 2009 Diabetes UK. *Diabetic Medicine,* 26, 560–565 Special Report | UK | X | X | X |
| 1. James J. “Diabetes specialist nursing in the UK: the judgement call? A review of existing literature” *EDN,* 2011 | UK | X | X | X |
| 1. Kerr, M. “Inpatient Care for People with Diabetes: The Economic Case for Change” *Insight Health Economics* November 2011 | UK |  | X |  |
| 1. Kousoulis AA, Patelarou E, Shea S, Foss C, Ruud Knutsen IA, Todorova E, Roukova P, Portillo MC, Pumar-Méndez MJ, Mujika A, Rogers A, Vassilev I, Serrano-Gil M, Lionis C1. “Diabetes self-management arrangements in Europe: a realist review to facilitate a project implemented in six countries.” *BMC Health Serv Res.* Oct 2;14:453. 2014 | Europe | X | X |  |
| 1. Lawal, M.. “The importance of diabetes specialist nurses” *Nursing in Practice.* [online] Nursinginpractice.com. 2018 | UK | X |  |  |
| 1. Loveman E, Royle P, Waugh N. “Specialist nurses in diabetes mellitus.” *Cochrane Database of Systematic Reviews*, Issue 2. 2003 Art. No.: CD003286. | Global |  | X |  |
| 1. Macmillan Cancer Support. “Specialist adult cancer nurses in England. A census of the specialist adult cancer nursing workforce in the UK” 2014 | UK | X |  |  |
| 1. Mahaffey K, Stanisstreet D, Ford M, Chapman L, Summerhayes B, Brown S, George S, Winocour P “Role of the diabetes inpatient specialist nurse in preventing hospital admission from A&E”, *Journal of Diabetes Nursing* Vol 16 No 2, 2012 | UK |  | X |  |
| 1. Modic M, Albert N, Nutter B, Coughlin R, Murray T, Spence J, Brosovich D. “Diabetes teaching is not for the faint of heart: are cardiac nurses up to the challenge?” *J Cardiovasc Nurs*. Nov-Dec 2009;24(6):439-46. | US | X |  |  |
| 1. National Diabetes Inpatient Audit (NaDIA) - 2016, Digital NHS | UK | X | X | X |
| 1. National Diabetes Inpatient Audit (NaDIA) – 2017, Digital NHS | UK | X | X | X |
| 1. National Diabetes Support Team Improving emergency and inpatient care for people with diabetes. 2008 | UK |  |  | X |
| 1. NHS England, National Diabetes Treatment and Care Programme, Introduction to and supporting documentation for VALUE BASED TRANSFORMATION FUNDING SITE SELECTION Five Year Forward View, December 2016 | UK | X | X | X |
| 1. NHS England, Patient Safety Alert Stage Three: Directive Improving medication error incident reporting and learning. 20 March 2014. | UK |  | X |  |
| 1. NICE “Quality Standards Programme Diabetes in Adults National Institute for Health and Clinical Excellence”, Centre for Clinical Practice. 2011 | UK |  |  | X |
| 1. Ross, A.J., Anderson, J.E., Kodate, N. “Inpatient diabetes care: complexity, resilience and quality of care”, *Cogn Tech Work* 16: 91. 2014 | UK | X | X |  |
| 1. Royal College of Nursing, “Specialist Nurses Make a Difference” RCN Policy Unit, Policy Briefing 14/2009 | UK |  | X |  |
| 1. Ryder B, Burbridge W, Braycotton L, Ryder B, Cull M, Davies P, De P, Basu A, Lee B. “Inpatient diabetes: do-it-yourself electronic referral system to support and enhance the Think Glucose project.”; *Practical Diabetes,* Jun 2014; 31(5): 194-196. 3p. ISSN: 2047-2897 | UK | X |  |  |
| 1. Sampson M, Crowle T, Dhatariya K, Dozio N, Greenwood R, Heyburn P, Jones C, Temple R, Walden E. “Trends in bed occupancy for inpatients with diabetes before and after the introduction of a diabetes inpatient specialist nurse service” *Diabetic Medicine*, September 2006, 23(9):1008-1015 Language: English. | UK |  | X |  |
| 1. Taylor CB, Miller NH, Reilly KR, Greenwald G, Cunning D, Deeter A, Abascal L. “Evaluation of a nurse-care management system to improve outcomes in patients with complicated diabetes.” *Diabetes Care*. Apr 2003;26(4):1058-63. | US |  |  | X |
| 1. Thompson DM, Kozak SE, Sheps S. “Insulin adjustment by a diabetes nurse educator improves glucose control in insulin-requiring diabetic patients: A randomized trial.” *Canadian Medical Association Journal* 1999;161(8):959–62. | Canada |  | X | X |
| 1. Vissarion B, Malliarou M Theofilou P, and Zyga S, “Improvement of Diabetic Patients Nursing Care by the Development of Educational Programs”, *Health Psychol Res*. Jan 13; 2(1): 931. 2014 PMCID: PMC4768559 PMID: 26973922 | Greece | X |  |  |
